# Perioperative predictors of early surgical revision and flap-related complications after microvascular free tissue transfer in head and neck reconstructions: a retrospective observational series

**DOI:** 10.1007/s00784-021-03864-1

**Published:** 2021-03-08

**Authors:** John-Patrik Burkhard, Jelena Pfister, Roland Giger, Markus Huber, Claudia Lädrach, Manuel Waser, Radu Olariu, Dominique Engel, Lukas M. Löffel, Benoît Schaller, Patrick Y. Wuethrich

**Affiliations:** 1 Department of Cranio-Maxillofacial Surgery, Inselspital, Bern University Hospital, University of Bern, CH-3010 Bern, Switzerland; 2 Department of Oto-Rhino-Laryngology, Head and Neck Surgery, Inselspital, Bern University Hospital, University of Bern, CH-3010 Bern, Switzerland; 3 Department of Anaesthesiology and Pain Medicine, Inselspital, Bern University Hospital, University of Bern, CH-3010 Bern, Switzerland; 4 Department of Plastic, Reconstructive and Aesthetic Surgery, Inselspital, Bern University Hospital, University of Bern, CH-3010 Bern, Switzerland

**Keywords:** Fluid overload, Free flap, Vasopressor, Revision surgery

## Abstract

**Objectives:**

The aim of this study was to determine the influence of perioperative fluid management and administration of vasopressors on early surgical revision and flap-related complications in free tissue transfer.

**Materials and methods:**

Intraoperative amount of fluid and of vasopressors, relevant perioperative parameters, and comorbidities were recorded in 131 patients undergoing head and neck microvascular reconstruction and compared with early surgical complications, defined as interventions requiring surgery after a flap-related complication, and/or other surgical problems in the operating room within 30 days after initial surgery. The relationship between perioperative variables for each revision category was determined using an optimized multiple logistic regression.

**Results:**

The administration of diuretics (*p*=0.001) as a treatment for perioperative fluid overload and the type of flap (*p*=0.019) was associated with a higher risk of early surgical revisions. Perioperative fluid overload (*p*=0.039) is significantly related to flap-related complications. We found no effect of intraoperative administration of vasopressors on early surgical revisions (*p*=0.8) or on flap-related complications (norepinephrine *p*=0.6, dobutamine *p*=0.5).

**Conclusion:**

Perioperative fluid overload is associated with higher risks of early surgical revision and flap-related complications. In contrast, the administration of vasopressors seemed to have no effect on either surgical revision rate or flap-related complications.

**Clinical relevance:**

In patients receiving microvascular reconstructions, a balanced fluid administration perioperatively and a targeted use of vasopressors should be the necessary strategy to reduce the complication rates in head and neck surgery.

## Introduction

Microvascular free tissue transfer is established in the head and neck area as a safe and reliable technique for reconstructing a large defect after extensive resection [1]. However, head and neck surgery involving free tissue transfer is complex and extensive and associated with an increased risk of complications related to high patient morbidity, resulting to prolonged hospital stays and higher costs [1–3].

In particular, free flap surgery requires sufficient blood circulation. Anesthesiologists face the difficult task of maintaining hemodynamic stability and tissue perfusion using crystalloids or colloids and the continuous i.v. administration of vasopressors or inotropes. However, the amount of fluid administered intraoperatively has been identified as an important predictor of poor results in free tissue transfer surgery, associated with a higher incidence of local and systemic complications [4–10]. In addition, there is considerable reluctance to use vasopressors due to hypothetical concerns about reduced graft perfusion during or after surgery, which creates additional limitations for anesthesiologists searching for evidence-based options for blood pressure control [11].

Various predictors of complications have been proposed, such as ASA (American Society of Anesthesiologists) physical status, previous attempted microvascular transplants, surgery duration, and higher tumor stages, but their importance still remains controversial [1, 5, 6, 9, 10, 12, 13].

The aim of this retrospective analysis was to assess the impact of intravenous administration of fluid, norepinephrine, and dobutamine intraoperatively on early surgical revisions (i.e., within 30 days) and flap-related complications after free tissue transfer. Furthermore, we aimed to investigate the influence of compounding factors.

## Material and methods

This retrospective observational study reports a consecutive case series from a single tertiary center. Ethical approval of this study was provided by the Ethical Committee of the Canton Bern, Switzerland (KEKBE 2019-01824), on January 28, 2020, and the need for informed consent was waived. The study was not registered. It conforms with the STROBE (Strengthening the Reporting of Observational Studies in Epidemiology) guidelines.

### Study population

We evaluated health-related data of 131 consecutive patients who received free tissue transfer surgery in the head and neck area. These included all malignant diseases of the oral cavity and the facial skin, osteoradionecrosis, and drug-induced osteonecrosis of the jaw. The patients were manually identified and selected from the clinic’s internal database. Relevant information and patient data were extracted from medical records, including paper charts and anesthetic protocols, and stored in the clinic database.

### Data collection and outcomes

Preoperative data collection included age, sex, type of pathology requiring free tissue transfer, and preoperative comorbidity (arterial hypertension, chronic obstructive pulmonary disease, diabetes mellitus, renal insufficiency, alcohol consumption, and smoking).

Intraoperative parameters included the type of surgical intervention, duration of surgery, flap type (osseous vs. non-osseous), total intraoperative administration of i.v. fluids (crystalloids, colloids, and amount of packed red blood cells), blood loss, and the total amount of vasopressors (norepinephrine and dobutamine) continuously administered.

Postoperative parameters included the lowest hemoglobin value within 5 days after surgery or before the decision for surgical revision if surgery was needed before postoperative day 5 (“nadir hemoglobin”), administration of i.v. furosemide as a treatment of fluid overload (“diuretics”), type of surgical revisions, and length of hospitalization.

The primary outcome was the incidence of surgical revision within 30 days after initial free tissue transfer surgery. We chose 30 days as it is expected to be related to the intraoperative and/or early postoperative management. Surgical revision was defined as all surgical interventions associated with the free flap transfer surgery, regardless of localization (donor, recipient, neck, tracheostomy, and flap-site), performed in the operating room. Flap-related complications included flap dehiscence combined with partial or total flap necrosis and anastomotic insufficiency or thrombosis. We aimed to identify independent risk factors for early surgical revision. In addition, we also performed a sub-analysis of surgical revision in patients who received osseous or non-osseous free tissue transfer.

### Intraoperative management

Standard monitoring consisted of a three-lead-ECG, pulse oximetry, and invasive (cannulation of the radial artery) blood pressure measurement. Liberal indication was given awake fiber optic nasal intubation (under continuous administration of remifentanil) if there was any uncertainty regarding airway safety. Induction medication consisted of propofol (2–3 mg/kg), fentanyl (1–2 μg/kg), and rocuronium (0.6 mg/kg) and thereafter bladder catheterization.

For the first part of the surgery, anesthesia was maintained with a combination of propofol and remifentanil until tracheotomy, followed by a switch to volatile anesthetics in combination with dexmedetomidine (0.3–0.5 mg/kg/h) and ketamine (20 mg bolus followed by 0.3 mg/kg/h), both to be terminated 30 to 60 min prior to the end of surgery along with reuptake of propofol and remifentanil as prophylaxis for postoperative nausea and vomiting.

Hemodynamic management (goal: systolic blood pressure ≥100 mmHg) was mainly carried out with Ringer’s lactate solution [14]. If the perfusion index of the pulse oximetry curve was >5 and urine output between 0.3 and 0.5 mL/kg/h, euvolemia was assumed, and continuous administration of low-dose norepinephrine (0.02–0.05 μg/kg/min) was initiated after consultation with the lead surgeon. Additional dobutamine (2–4 μg/kg/min) and colloids were initiated, if necessary. The transfusion threshold varied between 70 and 90 g/L hemoglobin.

Usually, a tracheostomy was performed and maintained to secure the patient’s airway during and after surgery. In the absence of tracheotomy, patients were extubated using a staged-extubation kit.

### Postoperative management

All patients were monitored overnight in the intensive care unit (ICU) before being transferred to the ward. Patients with non-osseous reconstruction were mobilized immediately, and those with osseous reconstruction after 5 days, according to the in-house regimen. For reconstructions within the oral cavity, nutrition was provided exclusively by a nasal or percutaneous stomach tube until wound healing was assured. A target systolic blood pressure above 100 mmHg was defined for sufficient flap perfusion [14]. Blood pressure drops were treated accordingly with volume administration (250–500 mL of crystalloids). Furosemide was administered intravenously when there were signs of overhydration (dyspnea, edema, weight gain). Flap control was performed visually and by Doppler sonography according to internal guidelines at defined times.

### Statistical analysis

Variables were compared between patients who needed an early surgical revision and those who did not. Data were expressed as median with interquartile range for continuous variables and frequencies for categorical ones. We performed exploratory landmark analyses for categorical data using the Fisher’s exact or the chi-square test, and for continuous data using the Kruskal-Wallis rank-sum test.

Factors were selected a priori based on their potential association with postoperative surgical revision and included alcohol consumption, early postoperative cardiac event, amount of intraoperative fluid volume (crystalloids, colloids, blood products) administered (in mL), intraoperative blood loss (mL), nadir hemoglobin (g/L), duration of surgery (min), and the total amount of norepinephrine (in μg) and dobutamine (in mg).

We first applied a univariable logistic regression of each predictor with the outcome and examined both the crude odds ratios and the odds ratios adjusted for “age” and “sex” of the patients. The univariable logistic regressions were followed by a multiple binary logistic regression model featuring all potential predictors. Again, crude and adjusted odds ratios were examined. The assumption of a linear relationship between the logit of the outcome variable and each continuous predictor used in this study was tested by creating a regression model with the logit as outcome and a set of predictors including the corresponding continuous predictor, its own natural logarithm, and an interaction term of each predictor and its natural logarithm. In all cases, the interaction terms were not statistically significant. Multicollinearity was examined by computing the variance inflation factor (VIF) for the predictors in the full binary logistic regression model. The corresponding values (from 1.1 to 2.7) suggested low levels of multicollinearity.

In terms of model selection, a parsimonious model was chosen with respect to known preoperative and intraoperative risk factors for a surgical revision due to the limited number of events and in order to avoid overfitting the regression. A stepwise backward selection procedure based on the Akaike information criterion (AIC) was used to identify independent risk factors for a surgical revision. Preoperative risk factors not associated with surgical revision in the final regression model and with a *p* value less than 0.05 were not included. The logistic regression model selected by the backstepping algorithm did not include the confounders “age” and “sex.” We show marginal effect plots for the final regression models to highlight the impact of each predictor on the outcome: to compute these plots, the values of a particular can vary while the other predictors are held constant (e.g., at their baseline levels or means) and the predicted probabilities of the outcome can be graphically displayed. In addition, we present nomograms of the final prediction models, which allow to estimate the probabilities of the outcome (e.g., probability of a surgical revision) for any given values of the predictors.

The fit of the multiple logistic regression models was assessed using the receiver operating characteristic-area under the curve (ROC-AUC) and an analysis of deviance (deviance check). A Monte Carlo cross-validation was performed to assess the predictive power of the two models on independent data that was not part of the model fitting procedure: the set of *n*=131 patients was randomly split into a training set (60% of all patients) and a test set (40% of all patients). The multiple logistic regression was subsequently performed using only the training data, and the computations of the ROC-AUC were based only on the test data. This procedure was performed 1000 times, resulting in a distribution of the ROC-AUC for each model.

A two-sided *p* less than 0.05 was considered significant. Analyses were performed using the R statistical package (R Foundation for Statistical Computing, Vienna, Austria, version 4.0.0).

## Results

We identified 131 consecutive patients who underwent head and neck free tissue transfer surgery between January 1, 2014, and September 9, 2019.

Overall, early surgical revisions occurred in 42/131 patients (32%). There were 5/42 (12%) with a radial forearm, 15/42 (36%) with an anterolateral thigh, 5/42 (12%) with a superficial iliac artery perforator, and 17/42 (41%) with a fibula free flap transfer (*p*=0.094). Revision occurred in 17/34 (50%) “osseous” free tissue transfers and in 25/97 (25.7%) “non-osseous” free tissue transfers (*p*=0.018). Median time to revision was 7 days [IQR 2.25–14] (*p*=0.001), but 9/42 revisions (21%) were performed within 24 h after initial surgery. Early surgical revision (42/131) and flap-related complications (25/131) were caused by anastomosis problems (6/42, 14.3%; 6/25, 24%), bleeding (9/42, 21.4%; 2/25, 8%), infection (5/42, 11.9%), dehiscence/necrosis (20/42, 47.6%; 14/25, 56% thereof 3 cases total flap necrosis), and seroma (2/42, 4.8%) respectively.

### Patient data

Patients who needed surgical revision were more prone to have preoperative chronic alcohol consumption (*p*=0.090), suffered more often from underlying cardiac diseases (*p*=0.007), and had surgeries of longer duration (*p*=0.033), increased intraoperative administration of crystalloids (*p*=0.023), increased intraoperative blood loss (*p*=0.049), and lower postoperative nadir hemoglobin values (*p*=0.008) (Table 1). The total intraoperative administration of norepinephrine (256 vs 192 μg, *p*=0.530) and dobutamine (25 vs 15 mg, *p*=0.903) did not differ between patients who needed surgical revision and those who did not (Table 2). In addition, the total intraoperative administration of norepinephrine (268 vs. 229 μg, *p*=0.652) and dobutamine (27 vs 16 mg, *p*=0.663) did not differ between patients with flap-related complications and those without.

**Table 1:** Baseline and clinical variables and type of pathology

Baseline characteristics	All	Surgical complications	No surgical complications	*p* values
	*n*=131	*n*=42	*n*=89	
Age, median [IQR], y	62.48 [54.76–71.88]	62.99 [56.7–71.89]	62.48 [53.7–71.78]	0.434
Sex				0.559
Male	85 (64.9%)	29 (69%)	56 (62.9%)	
Female	46 (35.1%)	13 (31%)	33 (37.1%)	
Alcohol use	57 (43.5%)	23 (54.8%)	34 (38.2%)	0.090
Tobacco use	80 (61.1%)	29 (69%)	51 (57.3%)	0.250
Hypertension	49 (37.4%)	14 (33.3%)	35 (39.3%)	0.565
COPD	24 (18.3%)	8 (19%)	16 (18%)	1.000
Diabetes mellitus	13 (9.9%)	5 (11.9%)	8 (6.1%)	0.755
Hemoglobin [g/L]	133 [123–133]	135 [121–141]	133 [123–140]	0.345
CKD classification				0.176
eGFR [mL/min] >90 (G1)	121 (92.4%)	40 (95.2%)	81 (91.0%)	
60–89 (G2)	6 (4.6%)	0 (0%)	6 (6.7%)	
30–59 (G3)	4 (3.1%)	2 (4.8%)	2 (2.2%)	
<30 (G4)	0 (0%)	0 (0%)	0 (0%)	
Preoperative radiotherapy	32 (24.4%)	6 (14.3%)	26 (29.2%)	0.256

*Abbreviations*: *CKD* chronic kidney disease, *COPD* chronic obstructive pulmonary disease, *eGFR* estimated glomerular filtration rate, *IQR* interquartile range, *SD* standard deviation, *y* year

**Table 2:** Surgical, intraoperative, and postoperative variables with status early surgical revision

Surgical and intraoperative characteristics	All	Surgical complications	No surgical complications	*p* values
	*n*=131	*n*=42	*n*=89	
Duration of surgery [min]	560 [489–644]	584 [519–701]	554 [480–625]	0.033
Type of reconstruction				0.018
Non-osseous	97 (74.0%)	25 (59.5%)	72 (80.9%)	
Osseous	34 (26.0%)	17 (40.5%)	17 (19.1%)	
Type of flap				0.094
Radial forearm flap	31 (23.7%)	5 (11.9%)	26 (29.2%)	
ALT	49 (37.4%)	15 (35.7%)	34 (38.2%)	
SCIP	15 (11.5%)	5 (11.9%)	10 (11.2%)	
Dorsalis pedis	1 (0.8%)	0 (0%)	1 (1.1%)	
Scapula	1 (0.8%)	0 (0%)	1 (1.1%)	
Latissimus dorsi	1 (0.8%)	0 (0%)	1 (1.1%)	
Fibula	33 (25.2%)	17 (40.5%)	16 (18.0%)	
Blood loss [mL]	600 [400–1000]	725 [500–1125]	600 [400–1000]	0.049
Intraop. i.v. fluid [total in mL]	5411 [4098–7307]	5823 [4573–7976]	4966 [4004–6861]	0.023
Fluid balance [mL]	3574 [705–8263]	3746 [2841–5769]	3542 [2788–4483]	0.314
Norepinephrine [total in μg]	229 [0–810]	256 [19–698]	192 [0–831]	0.530
Dobutamine [total in mg]	16 [0–44]	25 [0–42]	15 [0–44]	0.903
Postop Nadir Hb [g/L]	93 [87–101]	91 [85–94]	95 [88–103]	0.008
Length of hospital stay (days)	12 [10–16]	16 [11–22]	11 [9–14]	0.005

*Abbreviations*: *ALT* anterolateral thigh, *g/L* grams per liter, *Hb* hemoglobin, *kgBW* kilograms per body weight, *mcg* micrograms, *mg* milligrams, *min* minutes, *mL* milliliters, *SCIP* superficial circumflex iliac artery perforator

### Factors influencing early surgical revisions

**Fig. 1:**
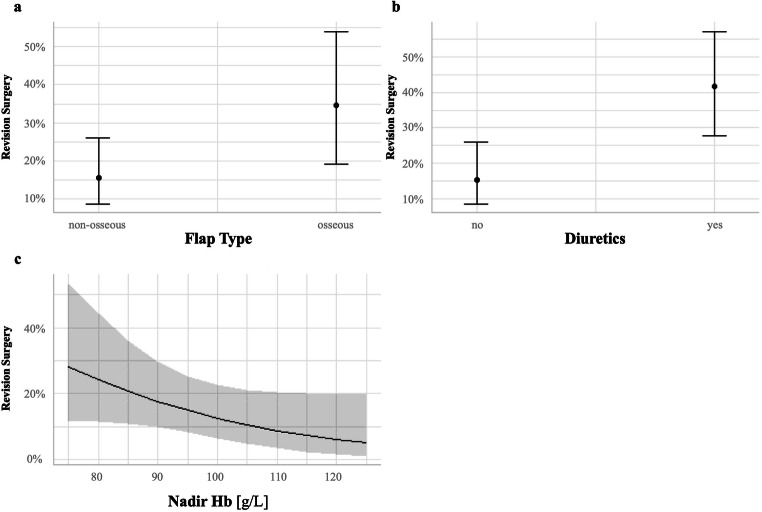
Marginal effect plots for the predictors included in the final model for early surgical revision. Each plot illustrates the effect of a particular predictor on the probability of a revision surgery while the other predictors are held constant. For example, panel **a** illustrates the probability of a revision surgery for the two categories of flap type while the predictors diuretics and nadir Hb [g/L] are held constant (at levels “no” for diuretics and a nadir Hb level of 94 [g/L]). *Abbreviations*: g/L, grams per liter; Hb, hemoglobin

**Fig. 2:**
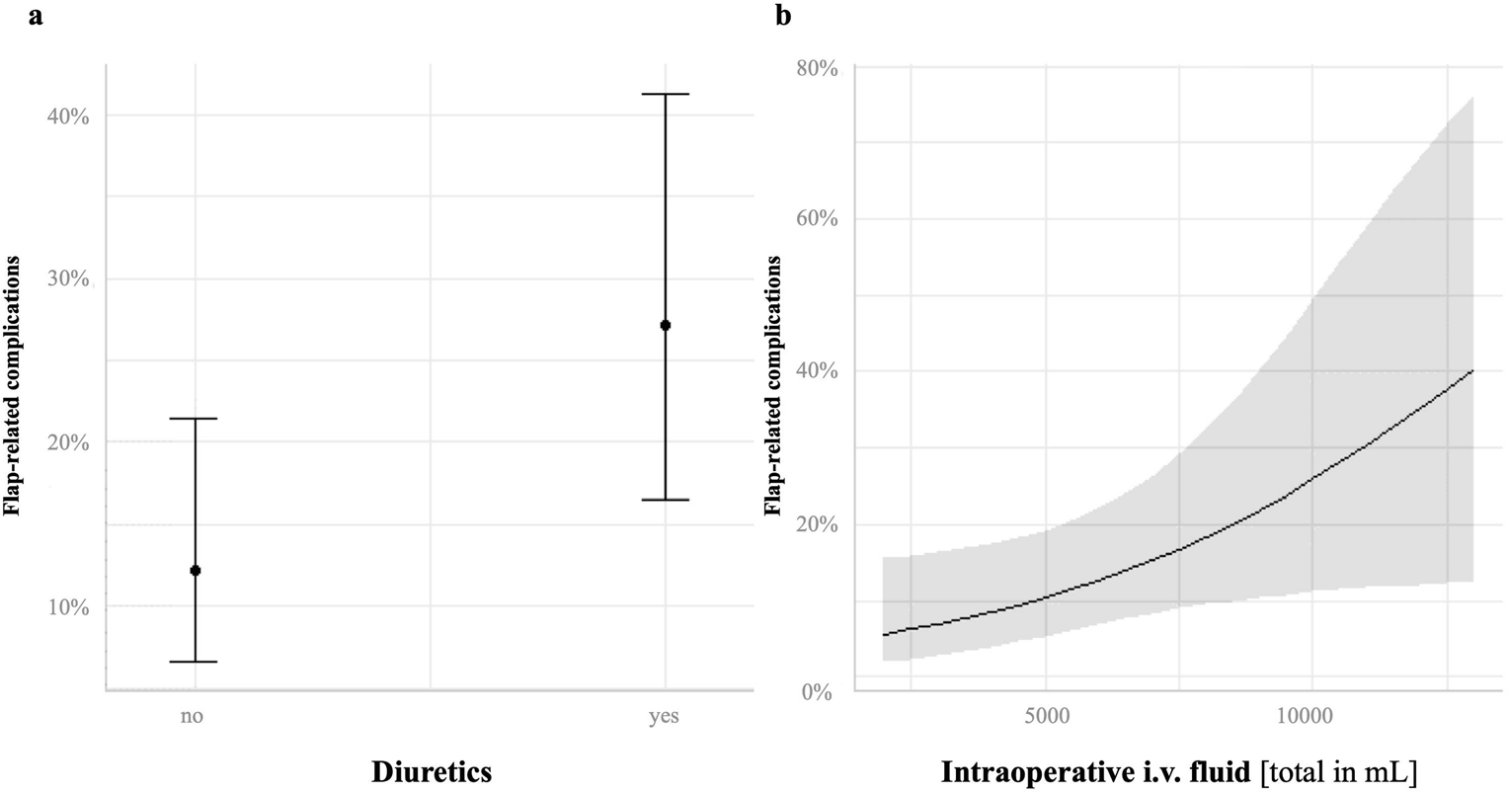
Marginal effect plots for the predictors included in the final model for flap-related complications. Panel **a** illustrates the probability of flap-related complications for the two categories of diuretics (holding the amount of intraoperative i.v. fluid a constant level), whereas panel **b** illustrates the probability of flap-related complications as a function of the amount of intraoperative i.v. fluid (while holding diuretics constant at the level “no”)

Effect plots are presented in Figs. 1 and 2. Regarding univariable logistic regression analysis, several parameters influenced early surgical revisions. Significantly higher revision rates were found for osseous reconstructions (2.88 [1.28, 6.55], *p*=0.011). In addition, diuretics (4.16 [1.94, 9.21], *p*<0.001) and nadir hemoglobin (0.95 [0.91–0.99], *p*=0.008) were seen as predictive values. Neither administration of norepinephrine nor administration of dobutamine (*p*=0.8) could be detected as a predictor. With regard to the influence of previous irradiation in the recipient area and the occurrence of early surgical revisions, no significance was shown in comparison to non-irradiated patients (0.40 [0.14–1.02], *p*=0.055).

Using backstepping multiple logistic regression analyses (optimized model), the variables “diuretics” (3.9345 [1.7556, 9.1400], *p*=0.001) and “flap type” (2.9020 [1.1973, 7.1826], *p*=0.019) remained significant. The detailed results can be seen in Table 3.

**Table 3:** **A** Regression models for early surgical revisions. **B** Sub-analysis of flap-related complications using multivariate regression

Dependent variable	Univariate			Multivariate (full model)			Optimized (step. AIC)		
*n*=131	OR	95% CI	*p* value	OR	95% CI	*p* value	OR	95% CI	*p* value
A									
Age at diagnosis (y)	1.01	0.98, 1.04	0.5						
Sex (male vs female)	1.31	0.61, 2.94	0.5						
Flap type (osseous vs non-osseous)	2.88	1.28, 6.55	0.011	2.9020	1.1973, 7.1826	0.019	2.9020	1.1973, 7.1826	0.019
Surgery duration (min)	1.00	1.00, 1.01	0.057						
Alcohol (yes vs no)	1.96	0.93, 4.15	0.075						
Diuretics (yes vs no)	4.16	1.94, 9.21	<0.001	3.9345	1.7556, 9.1400	0.001	3.9345	1.7556, 9.1400	0.001
Nadir Hb [g/L]	0.95	0.91, 0.99	0.008	0.9607	0.9163, 1.0042	0.084	0.9607	0.9163, 1.0041	0.084
Intraoperative i.v. fluid input [mL]	1.00	1.00, 1.00	0.091						
Blood loss [mL]	1.00	1.00, 1.00	0.053						
Norepinephrine [total in μg]	1.00	1.00, 1.00	0.8						
Dobutamine [total in mg]	1.00	0.99, 1.02	0.8						
Preoperative radiotherapy (yes vs no)	0.40	0.14, 1.02	0.055						
B									
Age at diagnosis (y)	1.01	0.97, 1.05	0.7						
Sex (male vs female)	1.92	0.74, 5.63	0.2						
Flap type (osseous vs non-osseous)	1.82	0.70, 4.57	0.2	1.7375	0.6248, 4.6706	0.3			
Surgery duration (min)	1.00	1.00, 1.01	0.2						
Alcohol (yes vs no)	1.86	0.78, 4.58	0.2						
Diuretics (yes vs no)	2.92	1.20, 7.34	0.018	2.4951	0.9905, 6.4794	0.054	2.6895	1.0887, 6.8733	0.034
Nadir Hb [g/L]	0.95	0.90, 1.00	0.031	0.9743	0.9208, 1.0276	0.3			
Intraoperative i.v. fluid [mL]	1.00	1.00, 1.00	0.021	1.0001	0.9999, 1.0004	0.3	1.0002	1.0001, 1.0004	0.039
Blood loss [mL]	1.00	1.00, 1.00	0.044	1.0003	0.9991, 1.0015	0.6			
Norepinephrine [total in μg]	1.00	1.00, 1.01	0.6						
Dobutamine [total in mg]	1.00	0.99, 1.01	0.5						
Preoperative radiotherapy (yes vs no)	0.97	0.33, 2.58	>0.9						

*Abbreviations*: *AIC* Akaike information criterion, *CI* confidence interval, *g/L* grams per liter, *Hb* hemoglobin, *i.v.* intravenous, *mcg* micrograms, *mg* milligrams, *mL* milliliters, *OR* odds ratio

### Factors influencing flap-related complications

We also identified independent factors specifically for flap-related complications following the similar regression models in the frame of a subgroup analysis. The following predictors could be detected in a final optimized model: diuretics (2.6895 [1.0887, 6.8733], *p*=0.034) and intraoperative fluid administration (1.0003 [1.0001, 1.0004], *p*=0.039). The administration of vasopressors could not be detected as a predictor. The detailed results can be seen in Table 3.

### Model significance

**Fig. 3:**
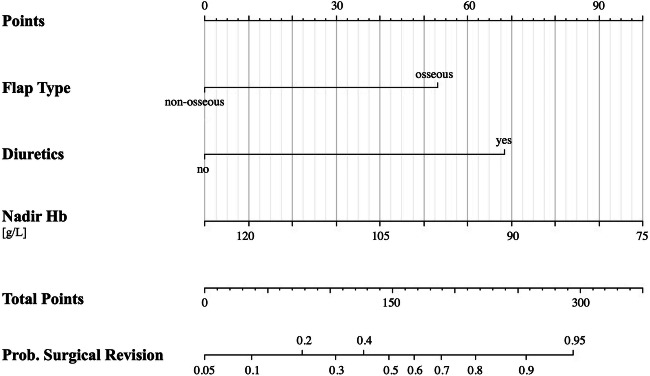
Nomogram predicting surgical revision based on the type of free tissue transfer. *Abbreviations*: g/L, grams per liter; Hb, hemoglobin; mL, milliliters

**Fig. 4:**
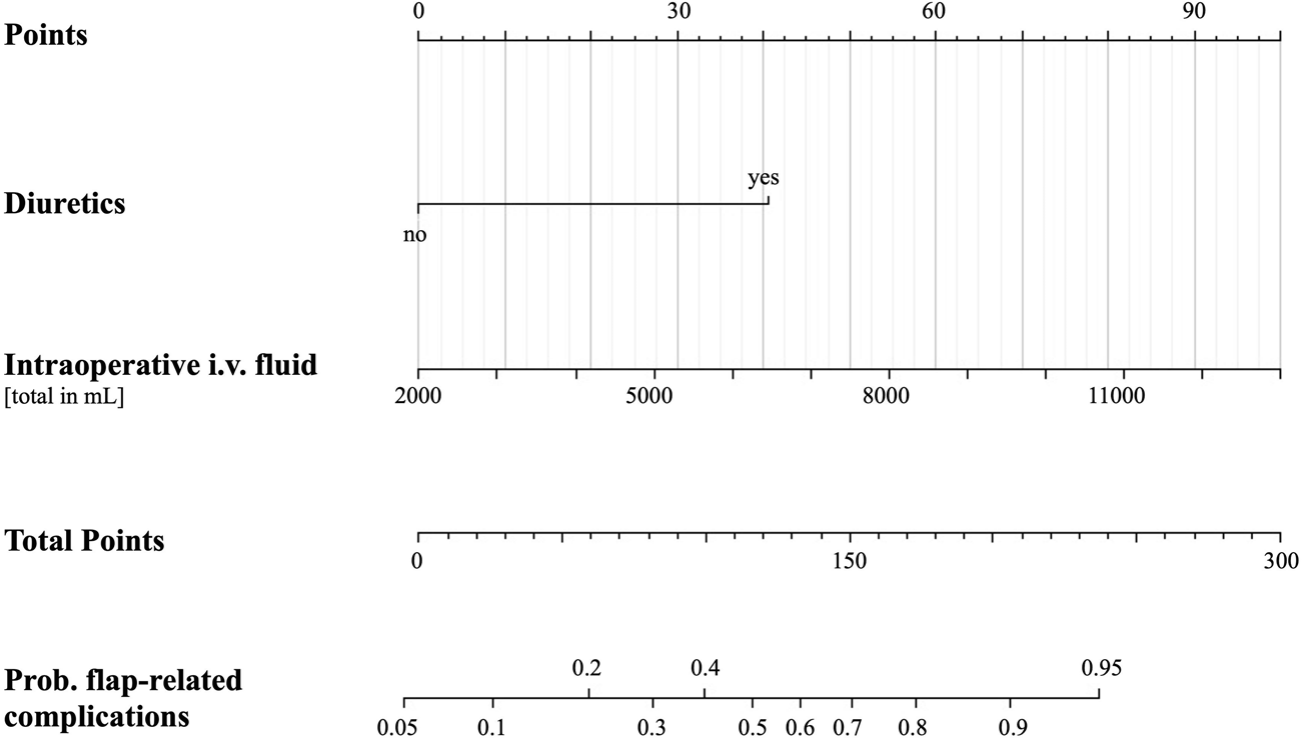
Nomogram predicting flap-related complications. *Abbreviations*: Hb, hemoglobin; mL, milliliters

The final optimized model (for early surgical revision) selection accuracy was good (ROC-AUC 0.77) and was also evaluated by the Monte Carlo cross-validation (ROC-AUC 0.77) for the full model. The predictive power (in terms of AUC) of the final optimized model without adjustment (with the confounders sex and age) showed the best performance, the AIC of the optimized, unadjusted model was lower 152 compared to the optimized, adjusted model (155). The increase in pseudo-*R*-squared metrics was also very modest (0.224 vs 0.212). These three observations confirmed that the best model was the optimized one without adjustment. Based on the independent variables depicted by the optimized multiple logistic regression model, we developed a nomogram for prediction of the surgical revision (Figs. 3 and 4).

## Discussion

This study provides evidence that postoperative fluid overload calculated on the administration of furosemide is associated with greater risks of early surgical revision and flap-related complications. In addition, excessive intraoperative fluid administration may increase flap-related complications. In our study, there was no observed association of early surgical revision or flap-related complications due to continuous administration of vasopressors. Surgical revisions were needed in 32% of the cases, and the overall failure rate of microvascular reconstructions was 2.29%, which is consistent with the current literature [15–21]. Furthermore, in around 12.9% of the revisions, the donor/recipient site, neck dissection site, or tracheotomy site was affected.

Hemodynamic stability—based on high cardiac output, normotony, hematocrit between 0.3 and 0.4 l/l, and urine output >1 mL/kg/h—is usually recommended for sufficient tissue perfusion and is a challenge for free tissue transfer surgery [22]. Suitable flap perfusion depends not only on vessel diameter, vessel tone, and an intact vessel barrier, but also on adequate perioperative fluid replacement [23–25]. In order to secure and maintain perfusion, the general indication for fluids or vasopressor administration is made.

Various studies [8, 10, 11, 26, 27] report that excessive volume of intraoperative crystalloids is predictive of postoperative complications. This is supported in our study, in which the administration of furosemide because of perioperative fluid overload was associated with an approximately fourfold increase in the risk of undergoing surgical revision within 30 days after the initial flap reconstruction [25, 28]. Patients with surgical revision received significantly more fluid intraoperatively and had greater blood loss with longer duration of surgery, a parameter albeit not detected as a predictor of revision or flap failure. More generous administration of fluid inevitably resulted in fluid accumulation in the interstitium. This resulted in tissue edema and suboptimal oxygen supply locally, a situation which has to be avoided in free tissue transfer. Therefore, the postoperative administration of furosemide acts against fluid overload and reduces interstitial edema. Similarly, longer periods of hypotension showed higher rates of flap failure. Part of this increased risk may be mediated by the effect of higher volume substitution in response to hypotension [9]. Both hypotension and large-volume fluid administration may lead to reevaluation of the role of vasopressors in free flap reconstruction.

The misconception that the use of vasopressors causes vasospasms in the vascular stem―leading to thrombosis, ischemia, and loss of the free tissue graft [29, 30]―is no longer supported by recent studies [9, 31–33]. The intraoperative application of vasoactive substances does not lead to a significant absolute increase in flap loss, as Monroe et al. [11] showed in a prospective study with 169 patients who received a free tissue graft in the head and neck region. To counteract hypotension induced by vasodilatation, low-dose vasopressors (e.g., norepinephrine 1–2 μg/kg/h) can be used instead of additional fluid administration. Numerous studies were unable to detect a negative effect of norepinephrine administration on the microcirculation [34–37]. These findings suggest that the use of vasopressors in microsurgery of the head and neck region is not a risk factor for developing flap-related complications per se.

To obtain sufficient tissue perfusion and normovolemia, blood loss and adequate blood transfusion must be considered. In this series, intraoperative fluid balance was similar between the groups, despite a significant increase in blood loss in the group with surgical revision. This illustrates good intraoperative hemodynamic management. However, as a low postoperative hemoglobin value was associated with surgical revision, a more differentiated fluid approach including blood transfusion should be considered at an earlier time point to support tissue oxygenation, rather than only perfusion.

Increased blood loss up to 1000 mL has been associated with higher risk of surgical revision, wound healing disorders, complication rates up to 27.8%, and failure rates of 6.5% in free tissue transfer in head and neck reconstruction [38–40]. Furthermore, low postoperative hemoglobin appears to be a significant predictor of flap-related complications and may be interpreted as a predictor of poor general condition, blood loss during surgery, and impaired oxygen delivery to the surgical wounds [41]. A hemoglobin value of <110 g/L seems to be an independent risk factor for postoperative surgical complications and prolonged hospitalization [42–44], just as lower values are a significant predictor of flap failure [45].

Our study confirms that intraoperative blood loss and lower postoperative hemoglobin are significantly associated with a risk of early surgical intervention. The critical hemoglobin value for blood transfusion in patients undergoing free flap surgery remains unclear. Kim et al. [39] recommend transfusion at a hemoglobin value lower than 87.5 g/L. Although blood transfusions have so far been used with great reluctance in microvascular surgery, recent studies show benefits [39]. A possible reason for this skepticism might be the resulting fluid overload, which clearly is a predictor of poor outcome, rather than blood transfusion per se. However, complications like flap failure, wound dehiscence, and wound edge necrosis at the interface of flap and autochthone tissue might signal a perfusion deficiency in the flap, whereas donor site seroma and tracheostoma bleeding are not related to flap perfusion problems.

This study is limited due to the patient population. To verify these results, prospective, controlled studies with higher patient numbers are necessary.

In order to integrate the central result of this study in clinical routine, the cooperation of surgeons and anesthesiologists performing free tissue transfers in head and neck surgery is of great importance. Balanced fluid administration perioperatively, avoiding persistently low hemoglobin values, and a targeted use of vasopressors may be the strategy needed to reduce complication rates in free flap head and neck surgery. Further prospective controlled studies focused on this topic are needed.

## Conclusion

Perioperative i.v. fluid overload requiring postoperative administration of furosemide was associated with higher risks of early surgical revision and flap-related complications in head and neck free tissue transfer. In addition, excessive intraoperative fluid administration may result in an increased number of flap-related complications. In contrast, the administration of vasopressors and inopressors per se seemed to have no effect on early surgical revisions and flap-related complications.
